# Energy dissipation evaluation of temperature swing adsorption (TSA) cycle based on thermodynamic entropy insights

**DOI:** 10.1038/s41598-019-53398-6

**Published:** 2019-11-12

**Authors:** Shuangjun Li, Shuai Deng, Li Zhao, Weicong Xu, Xiangzhou Yuan, Zhihao Guo, Zhenyu Du

**Affiliations:** 1 Key Laboratory of Efficient Utilization of Low and Medium Grade Energy (Tianjin University), Ministry of Education of China, Tianjin, 300350 China; 2 International cooperation research centre of carbon capture in ultra-low energy-consumption, Tianjin, 300350 China; 3 Department of Chemical and Biological Engineering, Korea University, Seoul, Republic of Korea

**Keywords:** Chemical engineering, Carbon capture and storage

## Abstract

The special report of the Intergovernmental Panel on Climate Change’s (IPCC) on global warming of 1.5 °C marks a critical point in climate negotiations, which emphasizes the importance to control the CO_2_ level in the atmosphere. The current technology cluster of CO_2_ capture is still energy-intensive which results in a substantial increase in costs, thus the efficient conversion among various forms of energy is the major topic of research. Considering that most of the existing research are primarily based on the viewpoint of energy conservation on a specific case study, the results thus could not be efficiently generalized as a condensed mechanism of energy dissipation. In this work, the entropy generation evaluation of a 4-step temperature swing adsorption (TSA) process was presented as a sample. The values and contribution distributions of various entropy generation in the thermodynamic cycle were calculated to evaluate the major energy dissipation. The results on contribution distribution of entropy generation and heat required were compared, the entropy generation distribution contributed by heat transfer decreases from 63.27% to 53.72% with internal heat recovery (IHR) method integrated. Thus the entropy generation saving potential of IHR method could be proved.

## Introduction

The Special Report on Global Warming of 1.5 °C has been released by the Intergovernmental Panel on Climate Change (IPCC) on 8th October 2018, which has modeling that the Global net human-caused emissions of CO_2_ would need to fall by about 45 percent from 2010 levels by 2030, reaching net zero around 2050^1^. It is obvious that the development of carbon capture technology would be needed to control the CO_2_ level in the atmosphere. Carbon capture could be executed with the application of three methods: a. Pre-Combustion Carbon Capture, b. Oxy-Combustion Carbon Capture and c. Post-Combustion Carbon Capture^2^, in which the Post-Combustion Carbon Capture has caught a lot of attention for that could be integrated with existing power plants.

A series of post-combustion technologies have been researched which include absorption, adsorption, membrane separation and so on, in which the absorption of CO_2_ by mature aqueous monoethanolamine (MEA) has been widely applied in demonstration project. But even for MEA absorption technology, the commercial utilization of which is still restricted by high energy consumption. Thus the concept of the thermodynamic cycle has been applied in MEA absorption research, which primarily focuses on the energy efficiency to confront with the energy-consumption problem^3^. And classic thermodynamic concepts such as COP and energy conversion efficiency have also been applied in the thermodynamic research on absorption technology^4^, compared the results of which with that in the ref.^5^, it could be found out that the energy consumption of absorption and adsorption are in the same level. This kind of method has also been applied in adsorption technology, Zhang^6^ has researched on the thermal energy required for regeneration of CO_2_-rich adsorbents, and concluded that the energy required can be greatly reduced by replacing the MEA technology with amine-based solid adsorbents capturing systems, due to the much lower heat capacity of solid adsorbents comparing to aqueous MEA and also the avoidance of evaporating a large amount of water in the regenerator. Thus the adsorption technology for carbon capture could be treated with energy-saving potential^7^. The thermodynamic carbon pump cycle has been proposed by Jiang^8^ to research on the energy-consumption of adsorption carbon capture technology, in which the minimum separation work and second law efficiency have been applied to evaluate the effect of operating parameters.

With the application of thermodynamic cycle concept in adsorption carbon capture technology, the energy required for adsorbents regeneration could be clarified. Zhao^9^ has researched on the comparative study of vacuum-pressure swing adsorption (VPSA) and pressure-temperature swing adsorption (PTSA) process based on the thermodynamic carbon pump theory, the effect of operating parameters on energy-efficiency was studied with the establishment of VPSA and PTSA thermodynamic cycle. Jiang^8^ has researched the 4-step temperature swing adsorption (TSA) process integrated with internal heat recovery (IHR) method, which primarily focuses on the improvement of exergy efficiency of the system. As effective performance improvement method of adsorption technology, the heat recovery method has been widely applied to reduce the energy consumption. Pan^10^ has researched three kinds of heat recovery methods which have been applied in recent adsorption refrigerators and contribution distribution of recovered heat but no entropy generation has been analyzed. Lu^11^ has proposed a heat pipe type adsorption refrigerator system, the COP of the system were evaluated with the combined recovery process in the view of the first law of thermodynamics. Zhang^6^ has researched the effect of the heat recover fractions on the degree of heat integration and the sensitivity analysis of cyclic parameters has been conducted to get the influential factors. It could be concluded that the current researches primarily focus on the amount of heat required in a thermodynamic cycle, while the essence of energy dissipation in the heat and mass transfer processes is still unclear, especially in the field of improvement method development.

With the thermodynamic carbon pump model proposed by Zhao^11^, the concept of entropy can be applied to research on the energy dissipation in the necessary step of a cycle, by which the guideline for the development of energy-saving method could be obtained. Myat^12^ has researched on performance analysis of adsorption cooling system using a thermodynamic framework with an entropy generation analysis, which research on the minimization of entropy generation in the system to lead to the maximization of the coefficient of performance, but the energy dissipation in each step is not mentioned. The concept of entropy generation has also been applied in the refs^13,14^, in which the dynamic performance of system was evaluated with such indicator.

In conclusion, the effect of heat recovery method on entropy generation is still not researched comprehensively as shown in the Table 1, the energy-saving potential of IHR method is primarily researched in the view of the first law of thermodynamics, the energy dissipation in each step of an adsorption thermodynamic cycle should be clarified. Thus in this conclusion, the entropy generation saving potential of such method was evaluated in the view of the second law of thermodynamics.

**Table 1:** The contents comparison of current studies and this work.

	Year	Authors	Energy required	Heat recovery method	Entropy generation	Reference
Existing studies	2012	Myat, *et al*.			√	^12^
	2015	Pan, *et al*.	√	√		^10^
	2015	Lu, *et al*.	√	√		^15^
	2016	Zhang, *et al*.	√			^6^
	2017	Zhao, *et al*.	√			^9^
	2018	Jiang, *et al*.	√	√		^8^
This work			√	√	√	

**Figure 1:**
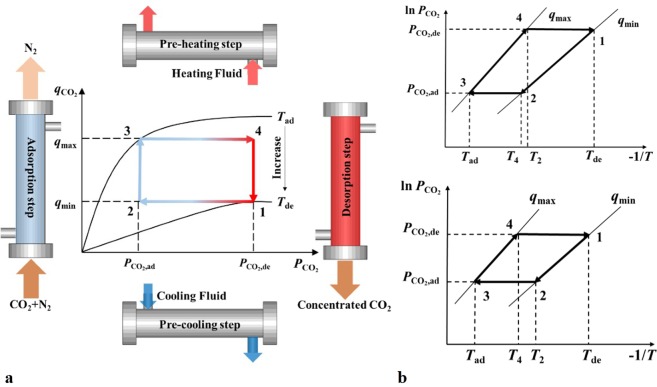
The schematic diagram of 4-step TSA process (**a**) and thermodynamic cycle established (**b**). The Clapeyron diagram has been widely applied in research on physical adsorption technology, such as adsorption refrigeration cycle^27^, which is also suitable for research on adsorption carbon capture technology like the 4-step TSA technology. As shown in the (**a,b**), step 1–2 is the pre-cooling process, in which the temperature of the adsorption reactor decreases from *T*_1_ (*T*_ad_) to *T*_2_ with no adsorbed amount changed. Step 2–3 is the adsorption process, the adsorbed amount increases with the CO_2_ partial pressure is *P*_ad_ in the atmosphere condition, and the adsorption heat is taken away by cooling fluid (such as cold water in *T*_ad_). Step 3–4 is the pre-heating process, in which the reactor bed is heated from *T*_3_ (*T*_ad_) to *T*_4_ with no adsorbed amount changed. And step 4-1 is the desorption process, the adsorbed amount decreases in this process with the CO_2_ partial pressure is *P*_de_ in the atmosphere condition, and the adsorption reactor is heated by heating fluid (such as hot steam in *T*_de_) continuously to *T*_1_(*T*_de_), then a new cycle starts. With characteristics of different adsorbents, the temperature in the end of pre-cooling process might be higher or lower than that in the end of pre-heating process as shown in the Fig. 2-b. The assumptions of the cycles researched are unrolled as follows: 1. The adsorbed CO_2_ is treated as looping fluid in this cycle. 2. The adsorbed amount of N_2_ is much lower than that of CO_2_ in the adsorbent, which can be neglected. 3. The temperature of adsorbent-adsorbate pair in the adsorption column is assumed as uniform. 4. The adsorption capacity of the adsorbent can be fully utilized in the whole thermodynamic cycle. e. The specific heat capacity of adsorbate is neglected for that is small enough as researched in refs^28,29^.

4-step TSA technology was researched in this work as a base case, the thermodynamic cycle established in the CO_2_ adsorption isotherms diagram was shown in the Fig. 1-a. This cycle could also be illustrated in the Clapeyron diagram, and two possible types of which as shown in the Fig. 1-b was researched in this work.

**Figure 2:**
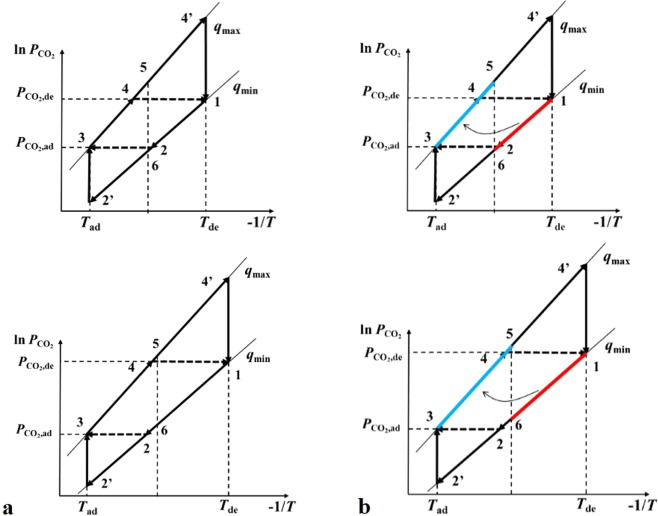
Decoupling of the thermodynamic cycle (**a**) and the schematic diagram of the cycle integrated with IHR method (**b**).

Considering that the heat and mass transfer exist in the steps 2–3 and 4-1 at the same time, the decoupling method could be applied to research on thermodynamic analysis more fluently. The decoupling of these two types of cycle was shown in the Fig. 2-a.

In the step 2–3, CO_2_ is adsorbed by adsorbent while the temperature decreases to *T*_ad_, the sensible heat of temperature variation and isosteric heat of adsorption are absorbed by the cooling fluid together. With the decoupling method, this step can be divided into two new isobaric steps: step 2-2′ and 2′-3. Thus the step 1-2-2′ is an isosteric step in all, in which the temperature decreases from *T*_de_ directly to *T*_ad_ and the step 2′-3 is an isothermal step, in which the amount of adsorbed CO_2_ increases in *T*_ad_.

The step 4-1 can be treated with a similar method, CO_2_ is desorbed from adsorbent while the temperature increases to *T*_de_, the sensible heat of temperature variation and isosteric heat of desorption comes from the heating fluid together. With the decoupling method, this step can also be divided into two new isobaric steps: step 4-4′ and 4′-1. Thus the step 3-4-4′ is an isosteric step, in which the temperature increases *T*_ad_ directly to *T*_de_ and the step 4′-1 is an isothermal step, in which the amount of adsorbed CO_2_ decreases in *T*_de_.

With internal heat recovery (IHR) method integrated, the thermodynamic cycle could be more complex, which could also be researched with the decoupling method. The IHR method has been widely applied in fluidized bed adsorption system^15^, which is also suitable for fixed bed adsorption system described in this paper to reduce the energy consumption. With the establishment of the thermodynamic cycle, the temperature variation of the adsorbent-adsorbate pair can be easily clarified to conduct the design of IHR method, which supply insight into the energy-saving potential.

The results in the ref.^6^ show that the sensible heat of adsorbent-adsorbate pair accounts for the primary part of energy consumption because of the vast temperature variation, thus the heat recovery method is suitable to be applied, and the IHR of sensible heat can reach 75% through special techniques^6^, which is researched in this paper. The application of IHR method can be unrolled in the decoupled schematic diagram of the 4-step TSA cycle with a simple case as shown in the Fig. 2-b. Two adsorption beds are applied to provide carbon capture capacity continuingly, thus a part of sensible heat of one adsorption bed in the pre-cooling step can be applied to supply the energy required of the other adsorption bed in the pre-heating step, which means that the sensible heat dissipated while the temperature of one bed decreases from *T*_de_ to *T*_6_ is recovered to supply the energy required to heat the other bed from *T*_ad_ to *T*_5_.

By circulating the working fluid in the circuit between the above two adsorption beds, sensible heat can be transported from the bed in the pre-cooling step to the other bed in the pre-heating step^16^ as shown in the Fig. 2. Considering that the heat transfer temperature difference is needed in the heat recovery exchanger, the minimum temperature difference between the working fluid in these two bed could be defined as the minimum temperature approach *T*_m_^17^. With the minimum temperature approach *T*_m_ defined, the *T*_5_ and *T*_6_ can be expressed as Eqs 1 and 2.

1 $${T}_{5}=[({T}_{ad}+{T}_{de})-{T}_{m}]/2$$

2 $${T}_{6}=[({T}_{ad}+{T}_{de})+{T}_{m}]/2$$

An ideal 4-step TSA thermodynamic cycle with IHR method could be researched when *T*_m_ is assumed as 0 K. Thus the value of *T*_5_ and *T*_6_ is equal in this ideal cycle, which can be expressed as shown in the Eq. 3:

3 $${T}_{5}={T}_{6}=({T}_{ad}+{T}_{de})/2$$

Thus with the decoupling method described, the entropy generation of heat and mass transfer could be calculated respectively in the 4-step TSA technology integrated with IHR improvement method.

## Results and Discussion

### Entropy generation analysis

**Figure 3:**
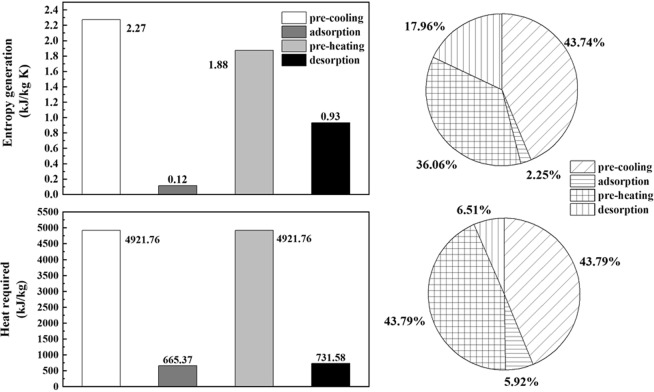
The amount and contribution distribution of entropy generation and heat exchanged.

Heat required analysis has been described in the ref.^8^, the numerical value and contribution distribution of entropy generation and heat required in the 4-step TSA technology were compared as shown in the Fig. 3. With the operating parameters listed in the Table 2 and the minimum temperature approach was assumed as 0 K, the entropy generation per kilograms captured CO_2_ of four steps mentioned above was calculated as illustrated.

**Table 2:** The operating parameters of the thermodynamic cycle (The pressure of surrounding is assumed as 1.0 bar).

Parameters	Values	Units
*T*_*ad*_	398	K
*T*_*de*_	298	K
*P*_*de*_	0.9	bar
*P*_*ad*_	0.15	bar

The adsorption capacity of adsorbent is primarily affected by temperature in a TSA cycle, thus the energy required in the pre-cooling and pre-heating steps account for the major part, which both contribute to 43.79%, the similar trend is also obtained in the ref.^17^. Thus such cycle can be integrated with IHR method for the potential of sensible heat recovery, which would be researched in this work as well. As for the heat required in the adsorption and desorption steps, which is needed to push the mass transfer, only account to a small part in the view of the first law of thermodynamics. But in the view of the second law of thermodynamics, the contribution of the desorption step is quite different as shown in the Fig. 3. The major irreversibility of the 4 steps can be obtained in this section, which contributed by heat transfer accounts to a dominant distribution though, the mass transfer irreversibility in the desorption step cannot be ignored.

### The effect of the IHR method

**Figure 4:**
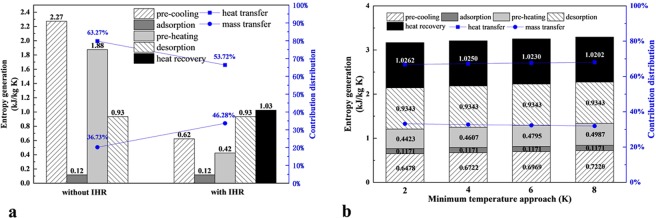
The amount and contribution distribution of entropy generation with or without IHR (**a**), and with different minimum temperature approach (**b**).

The IHR method has been unrolled above, and the effect of which on entropy generation was illustrated in the Fig. 4-a when the minimum temperature approach *T*_m_ is assumed as 0 K, the operating parameters are summarized in the Table 2. In the heat recovery step, the “cooling fluid” is applied to drop the temperature of one adsorption bed in the pre-cooling step, thus the “cooling fluid” is heated to a higher temperature and could be applied to increase the temperature of the other adsorption bed in the pre-heating step. Substitute the operating parameters into the Eq. 26, the entropy generation of the heat recovery step with per kilograms of adsorbate can be obtained. In results, the entropy generation decreases from 2.27 kJ/kg K to 0.62 kJ/kg K in the pre-cooling step, from 1.88 kJ/kg K to 0.42 kJ/kg K in the pre-heating step while that in the adsorption and desorption steps are constant. Thus the contribution distribution of entropy generation varies in the integrated technology, in which the heat and mass transfer account for 63.27% and 36.73% respectively without IHR method, 53.72% and 46.28% respectively with IHR method.

It can be concluded that the IHR method contributes an obvious distribution variation of entropy generation, the dominant role of entropy generation is still that caused by heat transfer though. Considering that the IHR method could be replaced by serial heat recovery or passive heat recovery method to further reduce the heat transfer irreversibility^10^, the method to reduce the mass transfer irreversibility should be considered such as pressure recovery method^18^.

Considering that the heat recovery time would vastly increases with minimum temperature approach *T*_m_ reduces, which would negatively influences the system performance. The effect of minimum temperature approach *T*_m_ on the entropy generation variation is also researched in this section, and the comparison of entropy generation in the whole cycle with *T*_m_ changes from 2 K to 8 K is illustrated in the Fig. 4-b. It can be concluded that the entropy generation of the whole system increases slightly with the *T*_m_ increases for the amount of heat recovered reduces. Considering that the minimum temperature approach cannot be eliminated in actual heat exchanger design, the effect of which on the irreversibility of the whole cycle should be learned.

### The effect of mainly operating parameters

**Figure 5:**
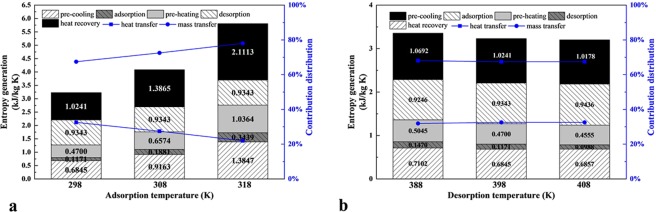
The amount and contribution distribution of entropy generation under different adsorption (**a**) and desorption (**b**) temperature.

The effect of mainly operating parameters on system performances of TSA technology, which include the minimum separation work and second-law efficiency, has been researched before^11^. However, how these parameters affect the entropy generation distribution in the whole cycle is still not clear. It is obvious that the TSA technology with IHR method is vastly influenced by temperature, thus the influence of adsorption and desorption temperature were researched in this section when the minimum temperature approach *T*_m_ was assumed as 5 K as shown in the Fig. 5, in which the assumed *T*_m_ is the common heat transfer temperature difference.

For the effect of adsorption temperature variation on the adsorption capacity in the cycle is larger, the entropy generation per kilograms adsorbed CO_2_ changes more obvious in the Fig. 5-a.

As shown in the Fig. 5-a, the entropy generation increases with the adsorption temperature. With the adsorption temperature increases from 298 K to 318 K, the entropy generation per kilograms CO_2_ captured increases from 3.23 kJ/kg K to 5.81 kJ/kg K which has changed nearly 80% with 20 K temperature increases. And the entropy generation contributed by heat transfer increases from 67.45% to 78.00% and that contributed by mass transfer decreases from 32.55% to 22.00% in total.

As shown in the Fig. 5-b, the entropy generation decreases as the adsorption temperature increases, it is obvious that the variation of the adsorbed amount is slight enough which has a slight effect on the variation of entropy generation. With the desorption temperature increases from 378 K to 398 K, the entropy generation per kilograms CO_2_ captured decreases from 3.36 kJ/kg K to 3.20 kJ/kg K. And the entropy generation contributed by heat transfer decreases from 68.06% to 67.44% and that contributed by mass transfer increases from 31.93% to 32.56% in total.

It can be concluded that though the temperature of adsorption and desorption directly influence the entropy generation caused by heat transfer, the entropy generation per kilograms adsorbed CO_2_ is primarily influenced by the amount of CO_2_ captured in the whole cycle. Thus the development of adsorbents to improve the adsorption capacity is still necessary to reduce the entropy generation in the 4-step TSA technology.

## Conclusions

In summary, an abstract thermodynamic cycle was focused on in this work, by which the basic characteristics of the 4-step TSA process integrated with the IHR method would be clarified. With the equilibrium state points connected by ideal processes, a thermodynamic cycle could be obtained, and the details of energy dissipation in the cycle would be clarified in the view of thermodynamics. With the application of decoupling method and adsorption potential theory, the energy dissipation mechanism in adsorption carbon capture technologies could be clarified with the calculation of entropy generation contributed by heat transfer and mass transfer respectively. Such a method could also be applied to a series of adsorption technologies as a general analysis, which relies on the basic characteristics of different technologies but not parameters of specific devices. Through the analysis with the same set of standards, a horizontal comparison between different technologies could be achieved.

## Methods

### Modeling of adsorption isotherms

The activated carbon mentioned in the refs^19,20^ is applied in this work as the adsorbent, the adsorption capacity of which in equilibrium is expressed as a function of temperature and pressure called isotherm model. The experimentally measured data in ref.^15^ are fitted using the Langmuir model as shown in the Eq. 4 in the adsorption condition.

4 $${q}_{ad}={q}_{m}bP/1+bP$$

In which the *q*_m_ and *b* are the parameters of the Langmuir model, more details of these could be found in the Table 3. And the Dubinin-Astakhov (D-A) model as shown in the Eq. 5 is applied to calculate the adsorbed amount in the desorption condition, the coefficients of which are also obtained from the ref.^19^.

5 $${q}_{de}=(\frac{{q}_{0}}{{v}_{a}})\exp (-{(\frac{A}{E})}^{n})$$

**Table 3:** The adsorption isotherm coefficients and thermodynamic properties of the adsorbent.

Adsorbent	Specific heat capacity *C*_p_ (kJ/kg K)	Temperature (K)	Langmuir model		D-A model		
			*q*_m_(mg/g)	*b*	*q*_0_(m^3^/g)	*n*	*E*(J/mol)
Activated carbon	0.825	298	500.00	0.14	8.09 × 10^−4^	0.67	3817.74
		308	434.78	0.15			
		318	400.00	0.15			

In which the *v*_*a*_ is the specific volume of the liquid adsorbate and *A* is the adsorption potential as calculated in the ref.^21^. The adsorption isotherm coefficients and thermodynamic properties of adsorbent are shown in the Table 3.

The working capacity *q*_w_ in the TSA cycle is defined in this paper, as shown in the Eq. 6, which unroll the amount of CO_2_ product obtained with per kilograms adsorbent in a cycle.

6 $${q}_{w}={q}_{ad}-{q}_{de}$$

In which the *q*_ad_ and *q*_de_ are calculated by Langmuir model and D-A model respectively, which represent the amount of adsorbed CO_2_ per kilograms adsorbent in the adsorption and desorption condition respectively.

### Modeling of entropy generation

The concepts of entropy and entropy generation are applied in this section, the entropy generation of primary steps in a 4-step TSA process is calculated clarify the major irreversibility in a cycle. With the research on IHR method represented in the section 2.3, the improvement of system performance and the energy-saving potential of such method could be unrolled with entropy generation analysis in this section.

The concept of entropy is defined by the second law of thermodynamics which states that the entropy of a closed system increases or remains constant. The entropy balance of a closed system is shown in the Eq. 7 ^12^:

7 $$T{S}_{ge}=T\Delta {S}_{system}+T\Delta {S}_{surrounding}$$

In which the ∆*S*_system_ and ∆*S*_surrounding_ represent the entropy variation of the system and surrounding respectively, and the system represents the adsorbate-adsorbent pair system and the surrounding represents the heating or cooling fluid in this work.

### The pre-cooling and pre-heating steps

Based on the decoupling of 4-step TSA cycle process, the entropy generation of the re-organized pre-heating step 3-4′ and pre-cooling step 1-2′ could be calculated as follows:

In the preheating step, the adsorbent-adsorbate pair system is heated by the heating fluid, thus the adsorbent-adsorbate pair system and the heating fluid are the system and surrounding in the Eq. 8 respectively. With the assumption of the heat source is infinite, the temperature of the system increases from *T*_ad_ to *T*_de_ and the temperature of the surrounding stays the same *T*_de_.

Thus the entropy generation of the pre-heating step with per kilograms adsorbent can be calculated as shown in the Eq. 8, in which the PAT (process-average temperature) was defined in the ref.^22^ as shown in the Eq. 9, and the entropy generation of the closed system per kilograms captured CO_2_ is shown in the Eq. 10.

8 $$\begin{array}{c}{S}_{ge,pre-heating}=\Delta {S}_{system}+\Delta {S}_{surrounding}\\ \,\,\,\,\,\,\,\,\,=\,{C}_{p}({T}_{de}-{T}_{ad})/PAT-{C}_{p}({T}_{de}-{T}_{ad})/{T}_{de}\end{array}$$

9 $$PAT=({T}_{de}-{T}_{ad})/\,\mathrm{ln}({T}_{de}/{T}_{ad})$$

10 $${s}_{ge,pre-heating}={S}_{ge,pre-heating}/{q}_{w}$$

In which *C*_p_ is the specific heat capacity of the adsorbent, the value of which could be found in the ref.^19^ as shown in the Table 3.

Be conducted with the similar method, the entropy generation of the pre-cooling step with per kilograms adsorbent can be calculated as shown in the Eq. 11, in which the PAT was shown in the Eq. 12, and the entropy generation of the closed system per kilograms captured CO_2_ is shown in the Eq. 13.

11 $$\begin{array}{c}{S}_{ge,pre-cooling}=\Delta {S}_{system}+\Delta {S}_{surrounding}\\ \,\,\,\,\,=\,{C}_{p}({T}_{de}-{T}_{ad})/PAT+{C}_{p}({T}_{de}-{T}_{ad})/{T}_{ad}\end{array}$$

12 $$PAT=({T}_{de}-{T}_{ad})/\mathrm{ln}({T}_{ad}/{T}_{de})$$

13 $${s}_{ge,pre-cooling}={S}_{ge,pre-cooling}/{q}_{w}$$

### The adsorption and desorption steps

Based on the decoupling of the 4-step TSA cycle, the mass transfer of CO_2_ between gas phase and adsorbed phase which in the adsorption and desorption steps are separated from heat transfer process, thus the entropy generation of the re-organized adsorption step 2′-3 and the re-organized desorption step 4′-1 can be calculated as follows:

With the assumption of these two steps react in the isothermal and isobaric condition, the energy conservation equation can be obtained based on the first law of thermodynamics as shown in the Eq. 14, in which no other work occurred besides volumetric work.

14 $$dU=\delta Q-pdV$$

With the assumption of the heat source in the surrounding is infinite, the entropy generation can be obtained as shown in the Eq. 15:

15 $$Td{S}_{ge}=Td{S}_{system}-\delta Q$$

Substitute the Eq. 14 into the Eq. 15, the Eq. 16 can be obtained:

16 $$Td{S}_{ge}=Td{S}_{system}-(dU+pdV)$$

With the definition of enthalpy as shown in the Eq. 17, the Eq. 18 can be obtained:

17 $$H=U+pV$$

18 $$dH=dU+pdV+Vdp$$

Considering that the adsorption and desorption steps react in the isobaric condition, the Eq. 18 can be simplified into Eq. 19:

19 $$dH=dU+pdV$$

With the definition of Gibbs free energy as shown in the Eq. 20 and the isothermal hypothesis based on the decoupling cycle, the Eq. 21 can be obtained.

20 $$dG=dH-TdS-SdT$$

21 $$dG=dH-TdS$$

Substitute the Eqs 19 and 21 into 16, the entropy generation can be obtained as shown in the Eq. 22:

22 $$d{S}_{ge}=-dG/T$$

The adsorption potential theory was applied in the analysis on adsorption and desorption steps, which treated the adsorption process as the isothermal compression process. In the adsorption step, the integral Gibbs free energy ∆*G*_ad_ change can be defined as the minimum isothermal work necessary to load the adsorbent to a given level, which can be calculated as shown in the Eq. 23 ^23–25^, thus the entropy generation of the closed system per kilograms captured CO_2_ *s*_ge,ad_ is shown in the Eq. 24.

23 $$\Delta {G}_{ad}=-RT{\int }_{0}^{{P}_{ad}}{q}_{ad}d\,\mathrm{ln}\,P/{q}_{ad}$$

24 $${s}_{ge,ad}=-\,\Delta {G}_{ad}/{T}_{ad}$$

In the desorption step, the Gibbs free energy change ∆*G*_de_ can be calculated based on the adsorption potential theory^26^, which can be calculated as shown in the Eq. 25, thus the entropy generation of the closed system per kilograms captured CO_2_ *s*_ge,de_ is shown in the Eq. 26.

25 $$\Delta {G}_{de}=-\,{R}_{g}T\,\mathrm{ln}(\frac{{P}_{sat}}{{P}_{de}})$$

26 $${s}_{ge,de}=-\,\Delta {G}_{de}/{T}_{de}$$

### The IHR step

In the IHR step, a closed circuit of heat transfer fluid connecting two adsorption bed is formed, and the heat can be transported from a bed in the pre-cooling step to the other bed in the pre-heating step by circulating the heat transfer fluid. Thus in this step, temperature of the system in the pre-cooling step decreases from *T*_de_ to *T*_6_ and temperature of the system in the pre-heating step increases from *T*_ad_ to *T*_5_, and the entropy generation per kilograms adsorbent can be calculated as shown in the Eq. 27, the entropy generation of the closed system per kilograms captured CO_2_ is shown in the Eq. 28.

27 $${S}_{ge,heatrecovery}={C}_{p}\,\mathrm{ln}({T}_{5}/{T}_{ad})+{C}_{p}\,\mathrm{ln}({T}_{6}/{T}_{de})$$

28 $${s}_{ge,heatrecovery}={S}_{ge,desorption}/{q}_{w}$$
